# Efficacy of intranasal fluticasone propionate and budesonide in management of allergic rhinitis—a prospective comparative study

**DOI:** 10.1186/s43163-021-00181-y

**Published:** 2021-11-27

**Authors:** P. Kiruba Shankari, Swathi Suresh, Rukaiah Fatma Begum

**Affiliations:** 1 Department of Pharmacy Practice, School of Pharmaceutical Sciences, Vels Institute of Science, Technology and Advanced Studies (VISTAS), Pallavaram, Chennai, Tamil Nadu India; 2 Department of Pharmacology, SRM College of Pharmacy, SRM Institute of Science, and Technology, Kattankulathur, Chennai, Tamil Nadu India

**Keywords:** Allergic rhinitis, Visual analogue scale, Absolute eosinophil count, Fluticasone propionate, Budesonide, Intranasal corticosteroids

## Abstract

**Background:**

Allergic rhinitis (AR) or Hay fever is a chronic inflammation of the nasal mucosa induced by IgE-mediated hypersensitivity due to exposure of various allergens. AR occurs as a response against these inhaled allergens that cause inflammation of nasal mucosal membranes. In this study, a reliable treatment for allergic rhinitis with maximum effectiveness and minimal side effects was assessed. This study compared the effectiveness of intranasal Fluticasone propionate (FUP) and intranasal Budesonide (BUD) in reducing the eosinophil count and in improving the nasal and ocular symptoms. This prospective study was conducted on 62 cases of allergic rhinitis and patients with mild-to-moderate allergic rhinitis were selected for the study. They were randomly divided into two groups; group I consists of 30 patients who received intranasal Fluticasone propionate aqueous spray, total daily dose of 200 μg (50 μg/spray) as 2 sprays in each nostril administered once daily, whereas the group II consists of 32 patients who received intranasal Budesonide aqueous spray, total daily dose of 400 μg/day (100 μg/spray) as 1 spray in each nostril administered twice daily.

**Results:**

Analysis on patient-based symptom scores revealed that both the groups showed statistically significant reduction in symptoms. Fluticasone propionate was found to be significantly more effective (*P* < 0.05) than Budesonide in reducing sneezing, nasal itching and majority of symptoms of individual symptom scores. Budesonide showed somewhat similar effect in reducing nasal blockage at 4 weeks of treatment.

**Conclusion:**

Clinically, both the drugs showed statistically significant improvement when compared to baseline, but Fluticasone propionate was superior at reducing nasal symptoms, ocular symptom and eosinophil count.

## Background

Allergic rhinitis (AR) or hay fever is a chronic inflammation of nasopharynx that occurs as a response against inhaled allergen exposure triggered by immunoglobulin E (IgE)-mediated inflammation of nasal membranes [1–3]. Allergic rhinitis is a symptomatic disorder triggered after inhalation of allergens such as house dust, mites, pollens, animal danders (cat and dog allergens, rodent), fungal, molds, yeast, insects and other allergens [4, 5]. The nasal symptoms include nasal congestion, rhinorrhea, sneezing and nasal itching, whereas ocular symptoms include watery eyes, burning, redness and itching eyes [6]. Allergic rhinitis was classified into three types; they are seasonal allergic rhinitis, perennial allergic rhinitis and episodic allergic rhinitis [7].

Allergens in the environment can synthesize allergen-specific immunoglobulin E production which interacts with B cells, T cells, mast cells, basophil and starts accumulation in nasal mucosa which involves a pathophysiology of allergic rhinitis and asthma. This receptor-bound accumulation due do exposure of allergens leads to the production of mediators such as histamines, leukotriene and other mediators that shows allergic responses as nasal and ocular symptoms [8]. To identify the allergens is an important step for diagnosis of allergic rhinitis, allergen avoidance and allergen-specific immunotherapy [9].

In the current standard, intranasal corticosteroids are best and effective first-line therapy recommended for allergic rhinitis in preventing and relieving nasal and ocular symptoms. In allergic process, corticosteroids have a major role in reducing mediators and inflammatory cells [10]. Most commonly, corticosteroids are used as nasal sprays to treat allergic rhinitis but in patients with moderate to severe symptoms, systemic treatment may be used [11]. In this study, we have conducted an observational study to compare the safety and efficacy of intranasal fluticasone propionate and intranasal budesonide in allergic rhinitis patients. Efficacy was assessed using visual analogue scale for total nasal and ocular symptoms [12]. Fluticasone propionate stimulates glucocorticoid receptors and has potent anti-inflammatory activities, which acts on inflammatory mediators responsible for inflammatory symptoms of allergic rhinitis [13]. Budesonide also has potent anti-inflammatory activity and reduces the hyper-reactivity of airways. Budesonide relieves symptoms caused by hay fever or other allergies [14].

In this study, a reliable treatment for allergic rhinitis with maximum effectiveness and reducing the risk of developing allergic asthma was assessed.

## Methods

### Patient selection

This clinical prospective observational comparative study includes 62 patients who were diagnosed with allergic rhinitis and they were randomly assigned into two groups conducted in ENT department, tertiary care hospital in Chennai, Tamil Nadu.

### Inclusion criteria

Patients aged 12 years and above diagnosed with the history of moderate to severe seasonal or perennial allergic rhinitis have symptoms like nasal itching, congestion, rhinorrhoea, sneezing and ocular redness, itching and watery due to rhinitis and have allergy to any of these—house mites, pollen, dust, animal dander, dairy products, molds, etc.—were included in the study. Diagnosis was based upon symptoms according to criteria proposed in the International Consensus of Rhinitis.

### Exclusion criteria

Patients suffering from upper or lower respiratory tract infection within the last 1 month prior to enrolment or those who received systemic or topical corticosteroids, herbal drugs, beta blocking agent within the previous month of enrolment, patients with asthma and COPD, nasal bleeding, obstructing nasal polyps, paranasal sinuses and pregnant women were excluded from the study.

This study was carried out in a tertiary care “ESIC Hospital” located at Chennai. The study protocol was explained to all subjects prior to enrolment which has been approved by institutional review board or ethics committee. Informed consent was obtained from the patients with proper information explained as per the Declaration of Helsinki. Patients’ details were collected (age, gender and past medical history) by interviewing the patient directly or through patient’s medical record to avoid confounding variables. Allergens which caused the allergic rhinitis was diagnosed by two main allergy tests such as the skin prick test in which the allergen is kept on surface of skin and pricked with a needle to know the substance which is allergic to patients and other is blood test to check the immunoglobulin (IgE) antibody in the blood to diagnose allergy.

The patients who were diagnosed with allergic rhinitis were randomly assigned into two groups; the first group (group I) received intranasal Fluticasone propionate aqueous spray (Flonase) with a total daily dose of 200 μg (50 μg/spray) as 2 sprays in each nostril administered once daily for 8 weeks. The second group (group II) received intranasal Budesonide aqueous spray (Budenase AQ) with a total daily dose of 400 μg/day (100 μg/spray) as 1 spray in each nostril administered twice daily for 8 weeks. The Eosinophil count, visual analogue total nasal symptom scores (VATNSS) and visual analogue total ocular symptom scores (VATOSS) were obtained from the patients to assess the reduction in severity of the symptoms, and the patients were reviewed at 4th and 8th week of the treatment. Patients were asked to review at 1-month interval to refill the prescription. The response to the intranasal steroids treatment was monitored by eosinophil count and visual analogue scale (VAS) assessment. The mean difference between baseline, 4th and 8th week of all the parameters were calculated and statistically analyzed.

### Visual analogue scale

**Fig. 1:**
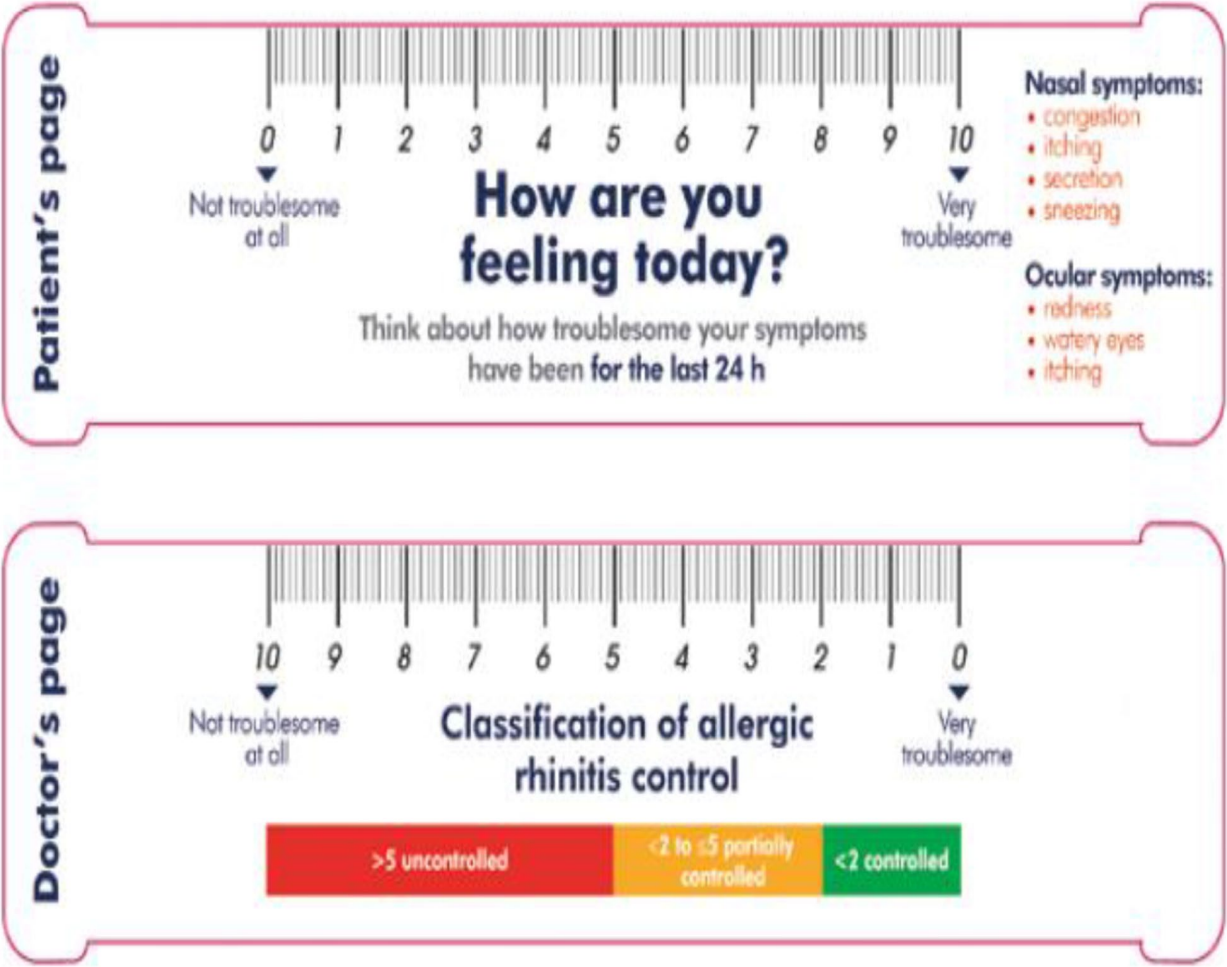
Visual analogue scale

Visual analogue scale for nasal and ocular symptoms can be measured using a horizontal scale having numbers from 0 to 10 with descriptions at its ends representing two extremes of feeling. Allergic rhinitis patients were asked to mark a point at the scale as per the severity of a single symptom or current status of disease control at baseline, 4th and 8th week of treatment for all the symptoms (Fig. 1) [15, 16]. The advantages of this scale are high resolution (minimal difference in disease severity can be distinguished), preferred by patients, reproducibility, uniform system of interpretation, routine use, linear scale, well suited for continuous features like AR symptoms, confirmed reliability and accuracy.

### Statistical analysis

Statistical analysis was performed by using the statistical software Graph Pad Prism with 95% confidence interval. Patient’s demographic characteristics analysis was done using MS Excel worksheet. Unpaired *T* test was used to evaluate mean change in VATNSS, VATOSS and Eosinophil count at baseline, 4 weeks and 8 weeks interval and to determine the significance. *P* value less than or equal to 0.05 was considered as statistically significant. One-way analysis of variance (ANOVA) was used in this study to assess the comparison of symptoms in both the groups.

## Results

### Baseline clinical characteristics

Sixty-two patients were enrolled in the study as per inclusion criteria and randomly assigned into 2 groups. Random assignment of 30 patients to intranasal fluticasone propionate taken as group I and 32 patients to intranasal budesonide taken as group II and these subjects completed the follow up of 8 weeks period. Demographics and baseline characteristics of 62 patients were noted and evaluated for primary and secondary parameters for 8 weeks baseline; clinical characteristics of patients were comparable values between the groups.

In the first group, 12 male (40%) and 18 female (60%) patients, their mean age was 37.1 ± 14.0 years and in the second group, 11 male (34.4%) and 21 female (65.6%) patients and their mean age was 38.6 ± 12.9 years. The mean and standard deviation (sd) of demographic parameters at baseline did not show any statistically significant difference (Table 1).

**Table 1:** Baseline clinical characteristics of patients with allergic rhinitis (AR)

Characteristics	Group I (***n*** = 30)	Group II (***n*** = 32)
**Age**	37.1 ± 14.0	38.6 ± 12.9
**Male**	12(40%)	11(34.4%)
**Female**	18(60%)	21(65.6%)
**IgE IU/mL**	417 ± 79.92	432 ± 71.45
**VATNSS**		
**Nasal congestion**	8.18 ± 1.25	8.50 ± 1.11
**Rhinorrhea**	7.32 ± 1.51	7.32 ± 1.72
**Sneezing**	9.06 ± 0.78	9.27 ± 0.69
**Nasal Itching**	8.69 ± 1.24	8.08 ± 1.41
**VATOSS**		
**Watery eyes**	7.59 ± 1.51	7.30 ± 1.53
**Redness**	7.78 ± 1.54	7.47 ± 1.55
**Burning**	6.90 ± 1.40	6.60 ± 1.39
**Itching eyes**	6.73 ± 1.42	6.65 ± 1.60
**Eosinophil count cells/mcL**	917.06 ± 249.14	904.37 ± 244.79

*VATNSS* Visual Analogue Total Nasal Symptom score, *VATOSS* Visual Analogue Total Ocular Symptom Score

Data are shown as mean ± standard deviation or number (%) as appropriate. Unpaired *T* test was used to determine significance

### Clinical efficacy

A total of 30 patients in group I and 32 patients in group II were evaluable and included in the efficacy analysis. Parameters considered in efficacy analysis were visual analogue total nasal symptom scores (VATNSS), visual analogue total ocular symptom scores (VATOSS) and Absolute eosinophil counts.

All the primary and secondary parameters showed statistically significant reduction in severity of symptoms in 8 weeks, proving clinical improvement in both the groups. However, group I showed statistical improvement of disease progression and reduction in severity of symptoms and eosinophil count.

### Visual analogue total nasal symptom score

Comparing the mean difference in nasal symptom scores between fluticasone propionate and budesonide during 8 weeks of treatment, FUP shows faster onset of action and statistically significant when compared to budesonide (Table 2).

**Table 2:** Efficacy outcomes of visual analogue total nasal symptom scores

Characteristics		FUP (***n*** = 30)	BUD (***n*** = 32)	***P*** value
**VATNSS**				
**Nasal congestion**	**Baseline**	8.18 ± 1.25	8.50 ± 1.11	0.29
	**4th week**	6.54 ± 1.35	6.27 ± 1.07	0.3
	**8th week**	4.9 ± 1.23	4.21 ± 1.07	0.02
	**P value**	**< 0.0001**	**< 0.0001**	
**Rhinorrhea**	**Baseline**	7.32 ± 1.51	7.32 ± 1.72	0.99
	**4th week**	5.23 ± 1.39	6.14 ± 1.63	0.02
	**8th week**	3.28 ± 1.28	4.93 ± 1.54	< 0.0001
	**P value**	**< 0.0001**	**< 0.0001**	
**Sneezing**	**Baseline**	9.06 ± 0.78	9.27 ± 0.69	0.26
	**4th week**	6.42 ± 0.93	8.05 ± 0.71	< 0.0001
	**8th week**	4.05 ± 0.62	6.82 ± 0.78	< 0.0001
	**P value**	**< 0.0001**	**< 0.0001**	
**Itching**	**Baseline**	8.69 ± 1.24	8.08 ± 1.41	0.07
	**4th week**	6.03 ± 1.19	6.76 ± 1.47	0.03
	**8th week**	3.83 ± 1.33	5.32 ± 1.48	< 0.0001
	**P value**	< 0.0001	< 0.0001	

Data are shown as mean ± standard deviation as appropriate. Unpaired *T* test was used to determine significance

*VATNSS* Visual Analogue Total Nasal Symptom Score, *FUP* Fluticasone propionate, *BUD* Budesonide

**Fig. 2:**
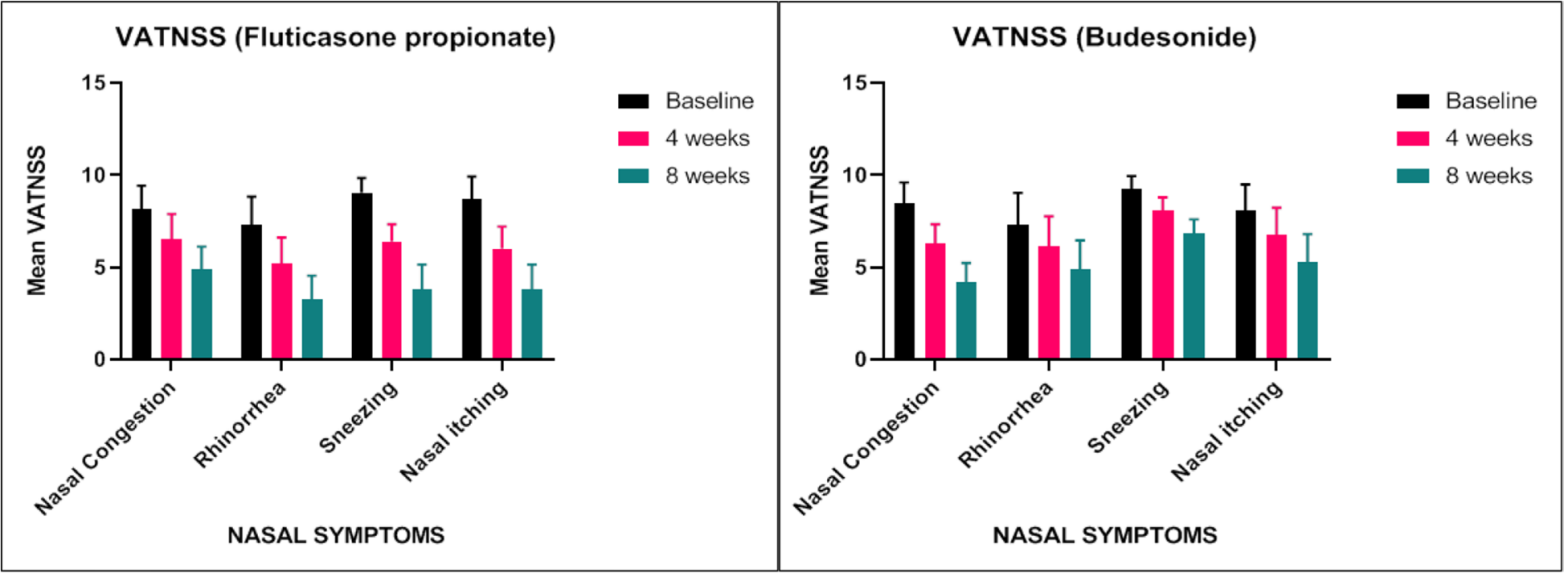
**A** Comparison of total nasal symptoms scores at baseline, 4th and 8th week in patients treated with Fluticasone propionate. **B** Comparison of total nasal symptoms scores at baseline, 4th and 8th week in patients treated with Budesonide

During the first 4 weeks of treatments, FUP significantly reduced the VATNSS compared with BUD. In terms of individual symptoms, FUP was significantly more effective than budesonide at reducing sneezing and nasal itching throughout the 8 weeks of treatment, and also significantly more effective in alleviating sneezing, rhinorrhea and nasal itching during 8 weeks of treatment (*P* < 0.001). The reduction in symptom scores for nasal blockage was similar in the FUP and BUD groups during weeks 1–8 (Fig. 2A, B).

### Visual analogue total ocular symptom scores

**Fig. 3:**
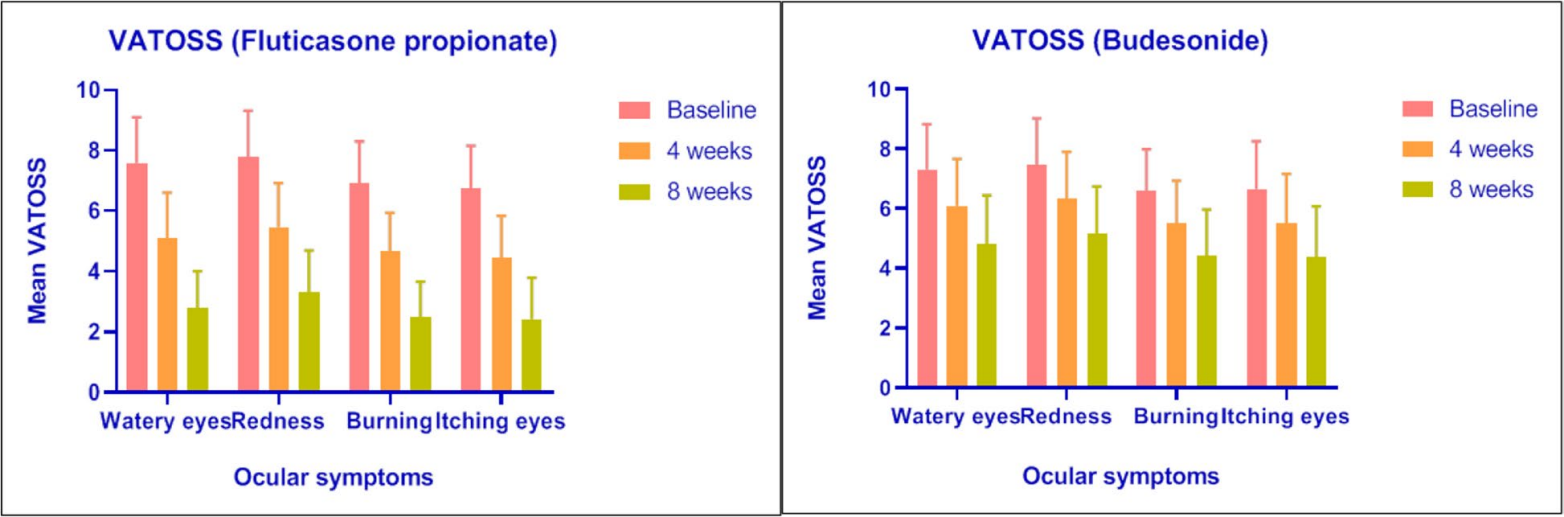
**A** Comparison of total ocular symptoms scores at baseline, 4th and 8^th^week in patients treated with Fluticasone Propionate. **B** Comparison of total ocular symptoms scores at baseline, 4th and 8th week in patients treated with Budesonide

Mean and standard deviation differ in ocular symptom scores between fluticasone propionate and budesonide during 8 weeks of treatment. During 8 weeks of treatment, FUP significantly reduced the VATOSS when compared with BUD. In terms of individual symptoms, FUP was significantly more effective than budesonide at reducing redness, burning, itching eyes and watery eyes throughout the 8 weeks of treatment (Fig. 3A, B).

The changes in FUP group were significantly greater than budesonide during weeks 1–4 (*P* < 0.0001) and weeks 4–8 (*P* < 0.0001). However, BUD group also showed significant difference of ocular symptoms from baseline to 8 weeks but BUD group shows less efficacy and onset of action when compared with FUP group (Table 3).

**Table 3:** Efficacy outcomes of visual analogue total ocular symptom scores

Characteristics		FUP (***n*** = 30)	BUD (***n*** = 32)	***P*** value
**VATOSS**				
**Watery eyes**	**Baseline**	7.59 ± 1.51	7.30 ± 1.53	0.45
	**4th week**	5.09 ± 1.51	6.09 ± 1.57	0.01
	**8th week**	2.80 ± 1.22	4.80 ± 1.64	< 0.0001
	**P value**	**< 0.0001**	**< 0.0001**	
**Redness**	**Baseline**	7.78 ± 1.54	7.47 ± 1.55	0.43
	**4th week**	5.46 ± 1.46	6.31 ± 1.59	0.03
	**8th week**	3.32 ± 1.37	5.14 ± 1.60	< 0.0001
	**P value**	**< 0.0001**	**< 0.0001**	
**Burning**	**Baseline**	6.90 ± 1.40	6.60 ± 1.39	0.4
	**4th week**	4.67 ± 1.26	5.49 ± 1.44	0.02
	**8th week**	2.49 ± 1.18	4.43 ± 1.53	< 0.0001
	**P value**	**< 0.0001**	**< 0.0001**	
**Itching eyes**	**Baseline**	6.73 ± 1.42	6.65 ± 1.60	0.83
	**4th week**	4.44 ± 1.40	5.50 ± 1.66	0.008
	**8th week**	2.43 ± 1.37	4.38 ± 1.69	< 0.0001
	***P*** **value**	**< 0.0001**	**< 0.0001**	

Data are shown as mean ± standard deviation as appropriate. Unpaired *T* test was used to determine significance

*VATOSS* Visual Analogue Total Ocular Symptom Score, *FUP* Fluticasone propionate, *BUD* Budesonide

### Eosinophil counts

In FUP group, the mean ± SD of eosinophil count at baseline was 917.06 ± 249.14 and decreased to 917.06 ± 249.14 and 418.23 ± 88.60 at 4th and 8th week of treatment respectively, which shows statistically highly significant. In BUD group, the mean ± SD of eosinophil count at baseline was 904.37 ± 244.79 and slightly reduced to 777.31 ± 231.48 and 606.15 ± 166.53 at 4th and 8th week of treatment respectively (Table 4).

**Table 4:** Outcomes of eosinophil count

Characteristics		FUP (***n*** = 30)	BUD (***n*** = 32)	***P*** value
**Eosinophil count cells/μL**	**Baseline**	917.06 ± 249.14	904.37 ± 244.79	0.84
	**4th week**	655.26 ± 186.59	777.31 ± 231.48	0.02
	**8th week**	418.23 ± 88.60	606.15 ± 166.53	< 0.0001
	***P*** **value**	< 0.0001	< 0.003	

Data are shown as mean ± standard deviation as appropriate

*FUP* Fluticasone propionate, *BUD* Budesonide

Unpaired *T* test was used to determine significance

**Fig. 4:**
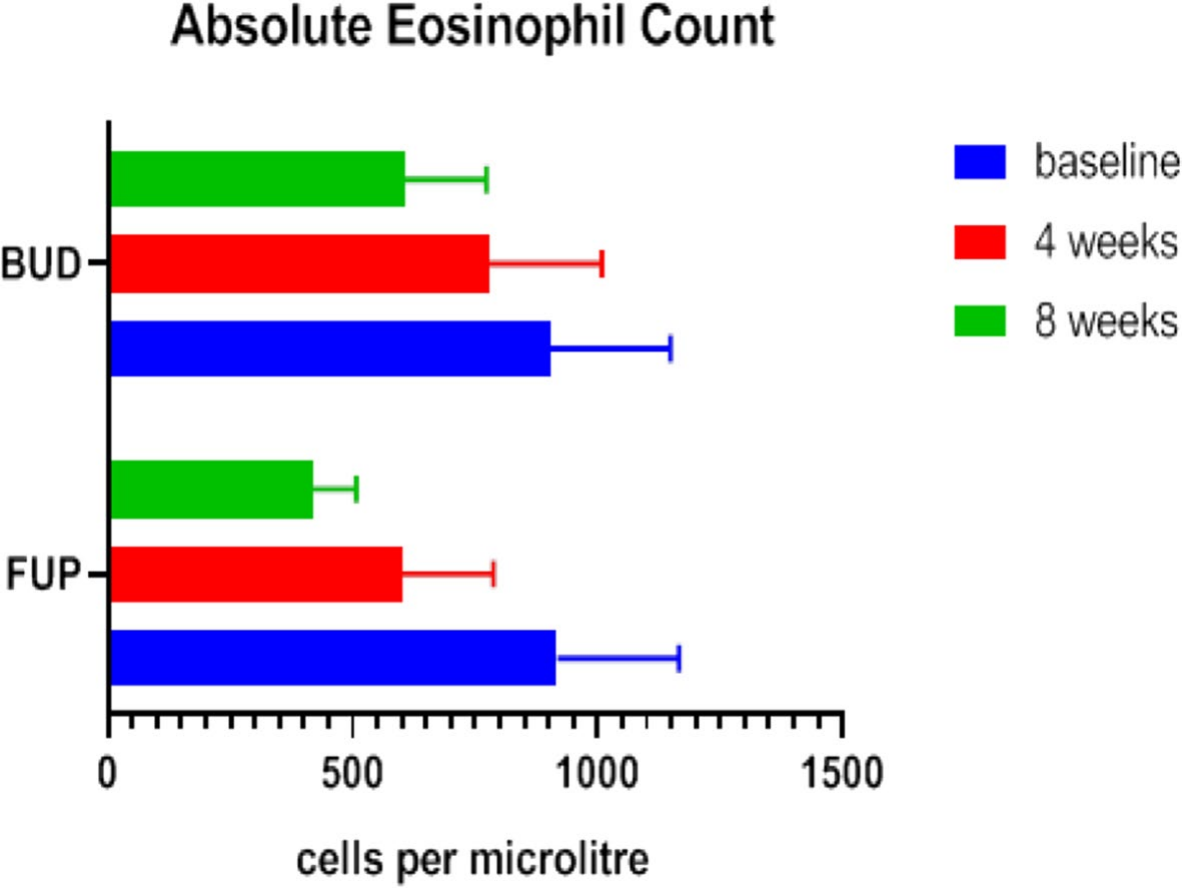
**A** Comparing the efficacy of intravitreal ranibizumab from baseline to 6 months in reducing central foveal thickness. **B** Comparing the efficacy of intravitreal triamcinolone from baseline to 6 months inreducing central foveal thickness

Mean reduction of eosinophil count from baseline over 8-week treatment period in both the group. Both Fluticasone propionate and budesonide showed statistically significant decrease in eosinophil count but fluticasone showed faster onset of action and greater immunologic improvement compared to budesonide. Hence, fluticasone propionate was found to be more efficacious than budesonide in reducing eosinophil counts (Fig. 4).

## Discussion

Allergic rhinitis or Hay fever is a chronic inflammation of the nasal mucosa induced by IgE-mediated hypersensitivity due to exposure of various allergens such as pollen, mold, animal dander, dust, mites, etc. AR occurs as a response against inhaled allergens that causes inflammation of nasal mucosal membranes [17].

The present study was carried out to assess the reduction in severity of symptoms and inflammatory cells in allergic rhinitis patients by comparing the intranasal corticosteroids such as Fluticasone propionate and Budesonide. The results of our comparative study demonstrate that Fluticasone propionate regularly once daily is more effective for the treatment of nasal and ocular symptoms in patients with allergic rhinitis than budesonide. This was proven by the study conducted by Lorenzo. G. D et al., which concluded that Fluticasone propionate administered monotherapy prevents eosinophil increase in nose during pollen season as measured in nasal lavage, whereas the use of mediator antagonists in combined therapy failed to produce [18].

A study conducted by Dellon E. S et al. concluded that a randomized clinical trial, initial treatment of Eosinophilic Esophagitis (EoE) with either oral viscous budesonide (OVB) or fluticasone multi dose inhaler (MDI) produced a significant decrease in esophageal eosinophil counts and improved dysphagia and endoscopic features [19]. Study by Tai C. J et al. has concluded that the patients with moderate to severe allergic rhinitis in patients who are sensitive to specific allergens during well define pollination season can provide greater immunologic effect by reducing serum IgE reaction to allergens [20]. The study on Allergic Rhinitis and its Impact on Asthma (ARIA) guidelines 2016 revision by Brozek J. L has recommended the measures of allergic rhinitis control using symptom scores, VAS Scores, quality of life scores and other several items. Using these recommendations, our study was carried out using VAS scores for assessing the severity of symptoms [21].

Recent study in allergy article by ARIA and European Academy of Allergology and Clinical Immunology (EAACI) proposed a questionnaire by Dutch ENT society to ARIA members all over the world. In the results of those questionnaire, 91.6% members of ARIA agreed to the question “currently nasal corticosteroid spray can be continued in hay fever season.” Hence, studies on intranasal corticosteroids can help in the current situation of Covid-19 patients with allergic rhinitis [22].

## Conclusion

Based on the above results, it is concluded that Fluticasone propionate therapy showed statistically significant benefits such as reduced Eosinophil counts and nasal and ocular symptoms when compared to Budesonide therapy. In addition, fluticasone propionate had a faster onset of action compared to budesonide in reducing eosinophil counts which were stimulated by IgE-mediated inflammatory cytokines. Hence, it is an effective treatment than budesonide in reducing sneezing, itching, rhinorrhea, ocular symptoms, and increased eosinophil counts of allergic rhinitis patient, reflecting its more potent topical anti-inflammatory activity.

### Limitations of the study

The main limitation of this study is that less number of patients included in the study and due to short period of study, side effects of the drug cannot be assessed and recurrence of the disease cannot be noted.

## Data Availability

Data and material are available upon request.

## Abbreviations

AR: Allergic rhinitis
BUD: Budesonide
FUP: Fluticasone propionate
IgE: Immunoglobulin E
VATNSS: Visual Analogue Total Nasal Symptom Scores
VATOSS: Visual Analogue Total Ocular Symptom Scores
VAS: Visual analogue scale
ANOVA: Analysis of variance
EoE: Eosinophilic esophagitis
OVB: Oral viscous budesonide
MDI: Multi dose inhaler
ARIA: Allergic Rhinitis and its Impact on Asthma
EAACI: European Academy of Allergology and Clinical Immunology
